# Antimicrobial Shape Memory Polymer Hydrogels for Chronic Wound Dressings

**DOI:** 10.1021/acsabm.2c00617

**Published:** 2022-10-18

**Authors:** Anand Utpal Vakil, Maryam Ramezani, Mary Beth B. Monroe

**Affiliations:** † Department of Biomedical and Chemical Engineering, BioInspired Syracuse: Institute for Material and Living Systems, Syracuse University, Syracuse, New York13244, United States

**Keywords:** shape memory polymers, polyurethanes, antimicrobial, hydrogels, phenolic acids, chronic wounds

## Abstract

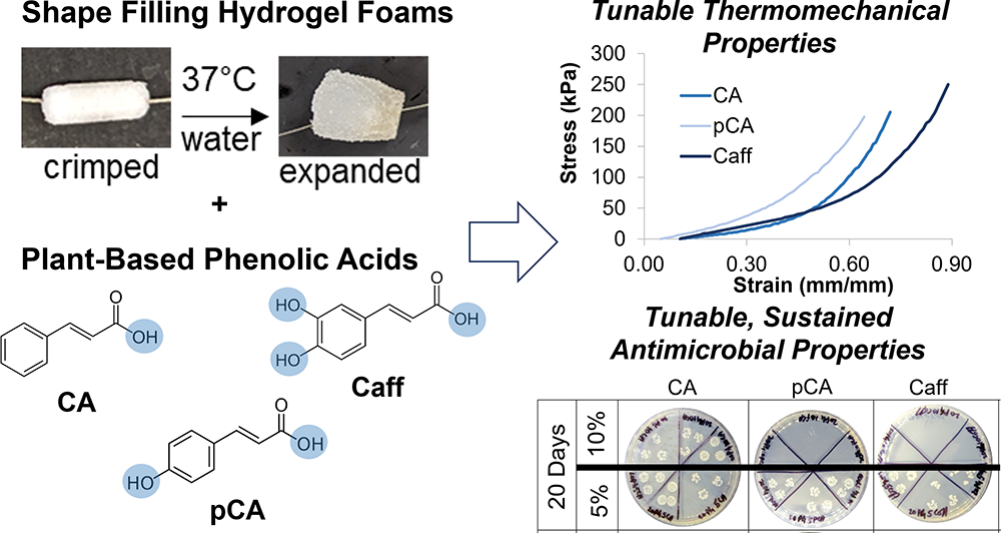

Chronic wounds can remain open for several months and have high risks of amputation due to infection. Dressing materials to treat chronic wounds should be conformable for irregular wound geometries, maintain a moist wound bed, and reduce infection risks. To that end, we developed cytocompatible shape memory polyurethane-based poly(ethylene glycol) (PEG) hydrogels that allow facile delivery to the wound site. Plant-based phenolic acids were physically incorporated onto the hydrogel scaffolds to provide antimicrobial properties. These materials were tested to confirm their shape memory properties, cytocompatibility, and antibacterial properties. The incorporation of phenolic acids provides a new mechanism for tuning intermolecular bonding in the hydrogels and corollary mechanical and shape memory properties. Phenolic acid-containing hydrogels demonstrated an increased shape recovery ratio (1.35× higher than the control formulation), and materials with cytocompatibility >90% were identified. Antimicrobial properties were retained over 20 days in hydrogels with higher phenolic acid content. Phenolic acid retention and antimicrobial efficacy were dependent upon phenolic acid structures and interactions with the polymer backbone. This novel hydrogel system provides a platform for future development as a chronic wound dressing material that is easy to implant and reduces infection risks.

## 1 Introduction

Wound care is a multibillion-dollar industry with $20 billion in annual costs.^1^ Wounds that fail to heal within the expected time frame (∼4 weeks)^2^ and do not respond to regular wound care treatment are considered chronic wounds.^3^ On average, chronic wounds affect approximately 2% of the American population annually.^4^ These slow-healing wounds can cause severe pain and discomfort due to prolonged inflammation, and they are highly susceptible to infection. In severe cases, nonhealing wounds require amputation. For example, in a study performed by a general hospital in Indonesia, it was found that 48% of diabetic foot ulcer patients required lower extremity amputation.^5^

Currently, chronic wound treatment options primarily involve cleaning the wound repeatedly, debridement, application of wound dressings, compression stockings/bandages, and antibiotics. More complex and expensive approaches include hyperbaric oxygen therapy, ultrasound and electromagnetic therapy, negative pressure wound therapy, and skin grafts. Repeated debridement and wound cleaning along with frequent bandage replacement can cause further discomfort and increased infection risks. Thus, improved wound dressing materials that effectively cover wounds and conform to wound walls to block entry of external bacteria could improve outcomes in chronic wounds. One such option involves moderately adsorbent hydrocolloids that can absorb small amounts of wound exudate and seal the wound.^6^ However, there is a possibility that hydrocolloids can trap the bacteria already present in the wound, rendering them unsuitable in cases where chronic wounds are already infected.

Alternatively, complete wound closure could be achieved by injectable hydrogels. Hydrogels are three-dimensional cross-linked polymer networks that can absorb large amounts of water (up to 10 times their dry weight). Hydrogels are typically made up of water-soluble polymers, and their cross-linked networks are resistant to dissolution in the body. Hydrogels can either be termed as permanent gels^7^ formed by a chemically cross-linked network, or physical gels^8^ formed by reversible physical interactions, such as hydrogen bonding or van der Waals forces. Hydrogels have a high potential to mimic the native skin extracellular matrix due to their high tunability and hydrated molecular structure.^9^ Both natural^10,11^ and synthetic^12,13^ hydrogels have been explored as potential candidates to treat skin defects. Some injectable hydrogels include Pluronics that comprises a triblock copolymer of polyoxyethylene (PEO) and polyoxypropylene (PPO). Pluronics have been used for continuous and controlled drug delivery at the implant site.^14,15^ Curcumin-loaded injectable Pluronic hydrogels were combined with gelatin to accelerate chronic burn healing and reduce scar formation.^16^ These composite hydrogels promoted the adhesion and proliferation of fibroblasts, indicating good cytocompatibility.

Another option for improved chronic wound filling is shape memory polymers (SMPs). SMPs are “smart” materials that can be deformed and stored in a temporary geometry and then triggered to return to their original shape upon exposure to an external stimulus, such as heat, pH, electrical impulse, or alternating magnetic field. SMP foams have been developed that utilize body temperature heating as a stimulus to trigger shape change.^17−19^ Here, we developed synthetic poly(ethylene glycol) (PEG)-based polyurethane SMP hydrogels as a potential wound dressing material. The high hydrophilicity of PEG could increase exudate absorption and subsequent swelling of hydrogels, allowing the hydrogels to maintain a moist environment, which may reduce pain.^20^ Antifouling properties of PEG could minimize adherence to underlying wounds.^21^ By combining PEG with a polyurethane network, shape memory properties can be achieved. Here, these hydrogels were prepared as porous foams. These foams can be compressed and stored into a constricted temporary geometry that enables easy application to wounds. Then, the hydrogel foams can expand back to their primary shape after implantation and heating to body temperature to fill up and seal irregularly shaped wounds.

In addition to effectively filling chronic wounds, one major concern with synthetic hydrogels involves reducing infection risks. To prevent antibiotic overuse, plant-based phenolic acids have proven beneficial as non-drug-based antimicrobials that are effective against multidrug resistant organisms (MDROs).^22,23^ Phenolic acids work by destabilizing bacteria cytoplasmic membrane, altering the permeability of the bacteria plasma membrane, inhibiting extracellular microbial enzymes, directly altering microbial metabolism, and/or depriving microbes of the substrate required for growth.^24^ Previously, phenolic acids were chemically cross-linked into polyurethane networks to provide antimicrobial scaffolds.^23^ To simplify scaffold fabrication and enable phenolic acid release over time, we focused on the physical incorporation of phenolic acids into these SMP hydrogels to provide antimicrobial scaffolds. In addition to antimicrobial properties, the hydroxyl groups on the phenol rings can potentially hydrogen bond to urethane linkages in these hydrogels to increase the net points and improve the shape memory properties. Increased shape fixity allows more stable storage in the temporary shape before implantation, and increased shape recovery enables rapid would filling upon implantation.

In this research, we prepared polyurethane SMP hydrogels as bulk (non-porous) scaffolds and foams with varying PEG molecular weights. Phenolic acids, including cinnamic acid (CA), p-coumaric acid (pCA), and caffeic acid (Caff), were physically incorporated into the hydrogel network at low and high concentrations. The resulting polymers were then characterized in terms of shape memory and antimicrobial properties. Retention of antimicrobial properties in the scaffolds and the surrounding media was characterized over one month of storage in phosphate-buffered saline.

## 2 Materials and Methods

### 2.1 Materials

Polyethylene glycol (*M*_n_ = 4000 Da and *M*_n_ = 6000 Da), triethanolamine (TEA), glycerol ethoxylate (*M*_n_ = 1000 Da), dibutyltin dilaurate (DBTDL), hexamethylene diisocyanate (HDI), granulated sodium chloride (NaCl), cinnamic acid (CA), *trans*-*p*-coumaric acid (pCA), caffeic acid (Caff), chloroform, dimethyl sulfoxide (DMSO), acetone, isopropanol, and Contrad solution were purchased from Fisher Scientific (Waltham, MA, USA). All chemicals used were reagent grade. PEG 4000, PEG 6000, and glycerol ethoxylate were dehydrated under vacuum overnight to remove any trace amounts of moisture before use. Sodium chloride granules were segregated using mesh screens to obtain fine granules within the 300–500 μm size range and dehydrated under vacuum overnight before use.

### 2.2 Hydrogel Synthesis

**Figure 1:**
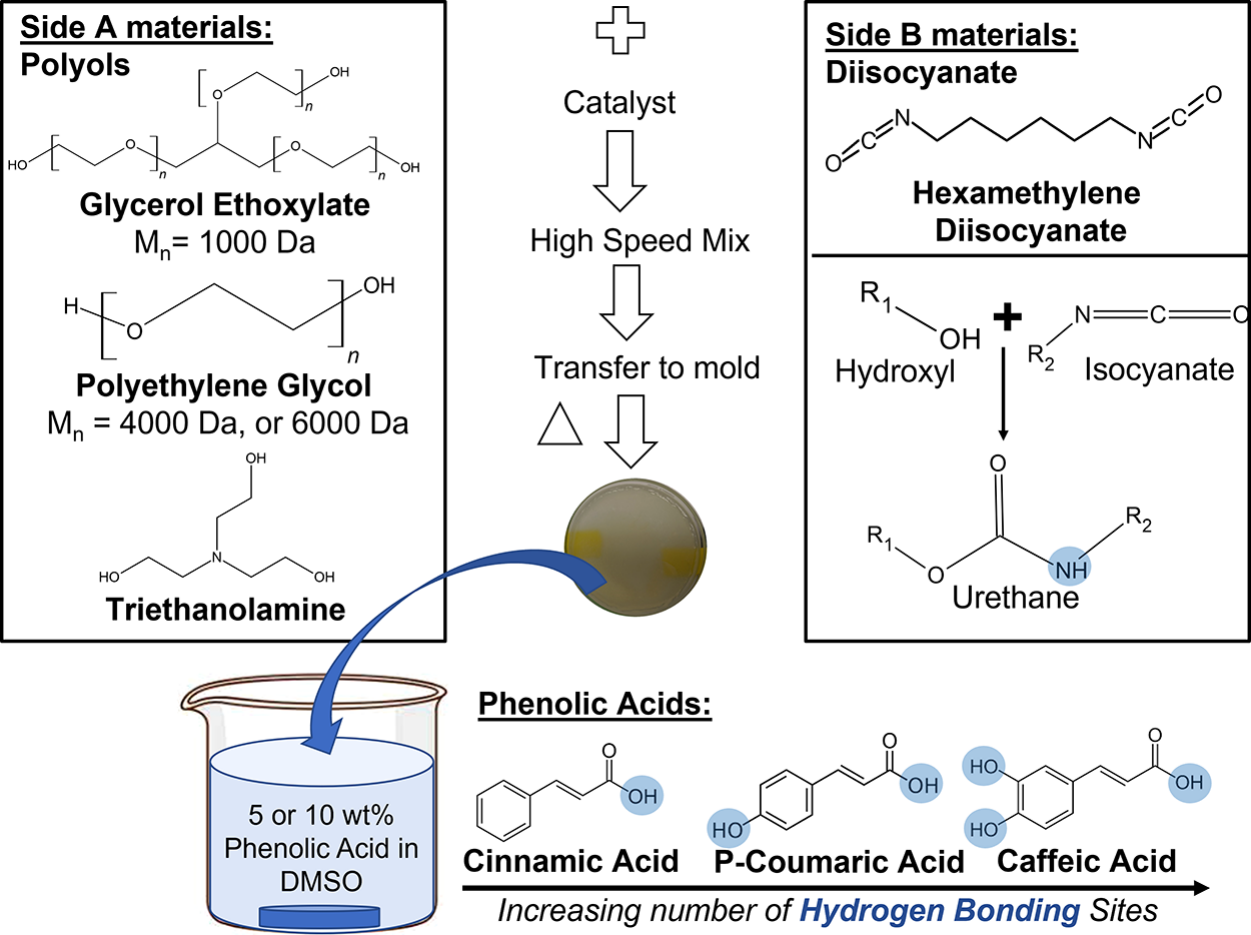
Synthesis of PEG 4000 and PEG 6000 hydrogels loaded with phenolic acids.

Two formulations were used as the basis on which the chemical and surface modification were studied. A combination of diols (PEG) and triols (TEA and glycerol ethoxylate) was used in each formulation. To ensure structural integrity and three-dimensional network formation, at least 80% of the hydroxyl groups were from the triols. The compositions of both formulations are shown in Table 1 on a weight basis that includes the amount of catalyst and monomers used. A schematic representation of hydrogel formation is shown in Figure 1. Side A materials represent the hydroxyl-containing components, and Side B materials denote isocyanate-containing components, which were combined to prepare a polyurethane hydrogel. All hydroxyl components were dissolved in chloroform before the addition of HDI. Then, the catalyst, DBTDL, was added. All additions were performed in a glovebox at <4% relative humidity under an inert nitrogen atmosphere. The reaction components were mixed in a speed mixer (Flacktek, Landrum, SC, USA) at 3500 rpm for 30 s. Reaction mixtures were poured onto 90 mm diameter polystyrene Petri dishes lined with a Teflon liner to form hydrogel films.

**Table 1:** Reactive Components of Synthesized Base Hydrogel Compositions in wt % with Resulting Gel Fraction and Swelling Ratios^a^

sample name	HDI	PEG 4000 (*M*_n_ = 4000 Da)	PEG 6000 (*M*_n_ = 6000 Da)	TEA	glycerol ethoxylate (*M*_n_ = 1000 Da)	DBTDL	gel fraction (%)	swelling ratio
PEG 4000	14.38	61.55	0	3.06	20.51	0.49	97.7 ± 0.3	1.92 ± 0.02
PEG 6000	10.92	0	70.16	2.32	15.6	0.99	96.6 ± 0.4	2.57 ± 0.02

a Mean ± standard deviation displayed. *n* = 3.

A subset of reaction mixtures was combined with sodium chloride granules before speed-mixing to form 70% porous (volume basis) foams. To ensure 70% porosity, the total volume of monomers was first estimated based on the density of each monomer. Then, the volume of NaCl added to each formulation was measured using a graduated cylinder to be 70% of the total monomer volume. Both films and foams were allowed to react in an oven for 24 h at 50 °C to ensure complete reaction and then vacuum-dried under −1015 mbar at 40 °C to remove excess chloroform from the network. Foams were stored in water for 48 h with the water changed at 24 h to wash out NaCl and provide open pores. Hydrogel films and foams were washed with water twice, 20% Contrad, isopropanol, and then acetone to remove catalysts and unreacted monomers. All washing volumes were 20 times the volume of samples. After washing, samples were dried overnight under a vacuum. Phenolic acids (CA, pCA, and Caff) were physically incorporated into the hydrogel network by soaking the hydrogels in 5 and 10 wt % phenolic acid solutions in DMSO overnight at 50 °C. Samples were then dried for 72 h under a −1015 mbar vacuum at 40 °C to ensure complete removal of DMSO. Samples were cut in required shapes for specific testing as described in the following sections.

### 2.3 Spectroscopic Analysis

The surface chemistry of dry hydrogel films was analyzed using a Nicolet i70 attenuated total reflectance (ATR)-Fourier transform infrared (FTIR) spectrometer (Fisher Scientific, Waltham, MA, USA) at 4 cm^–1^ resolution using OMNIC software (Fisher Scientific, Waltham, MA, USA). An average of 16 scans was used to generate a spectrum to confirm the physical absorption of phenolic acids onto the hydrogels.

### 2.4 Gel Fraction and Swelling Ratio

Post synthesis and before washing, 6 mm diameter punches were cut from hydrogel films and dried under vacuum at −1015 mbar at 40 °C for 24 h to remove any chloroform used as a solvent. For gel fraction measurements, the samples were weighed (initial dry weight) and placed in chloroform at 50 °C for 24 h to wash out any unreacted components. The washed samples were then vacuum-dried again at −1015 mbar and 40 °C and weighed again (dried sample weight) to measure gel fraction according to eq 1. In parallel, a second set of dried samples was weighed, placed in water at 50 °C for 24 h, and weighed again in the wet state to measure the swelling ratio according to eq 2.
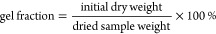
1
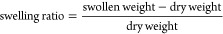
2

### 2.5 Phenolic Acid Loading

To measure phenolic acid loading, cylinders were weighed in the dry state after gel fraction measurements (i.e., after removing unreacted components) and then loaded with phenolic acids as described in section 2.2. Samples were dried under vacuum again, and the difference in dry masses before and after loading was taken as the mass of physically incorporated phenolic acids.

### 2.6 Thermal Analysis

Thermal analysis was performed on hydrogel films before and after phenolic acid incorporation. A thermogravimetric analyzer (TGA Q500, TA Instruments, Newcastle, DE, USA) was used to identify the temperature at which 3% mass loss occurs by heating 10 mg of the dry sample across a temperature range from 0 to 600 °C at 10 °C/min, shown in Figure S1 in the Supporting Information. This temperature was used as the upper limit at which samples were heated to identify their melting temperatures (*T*_m_) using a differential scanning calorimeter (DSC Q200, TA Instruments, Newcastle, DE, USA). Dry sample slices (3 to 5 mg) were loaded in t-zero aluminum pans. During the analysis, samples were equilibrated at −60 °C, kept isothermally for 2 min, heated to 100 °C at 10 °C/min, kept isothermally for 2 min, cooled to −60 °C at −10 °C/min, kept isothermally for 2 min and heated back to 100 °C at 10 °C/min. The *T*_m_ was measured as the endothermic peak minima temperature during the second heating cycle.

### 2.7 Shape Memory Properties

A dynamic mechanical analyzer (DMA Q800, TA Instruments, Newcastle, DE, USA) was used in controlled force mode to measure shape fixity and shape recovery ratios as an indication of the overall shape memory behavior of hydrogel films before and after the physical incorporation of phenolic acids. Samples (n = 3) were cut from the prepared hydrogel films using a dog bone punch according to ASTM D638 Type IV (scaled down by a factor of 4; length: 6.25 mm, width: 1.5 mm). The samples were heated to 60 °C and kept isothermally for 2 min. Then, a controlled force was applied to stretch the samples to a 40% strain at 0.03 N/min. The maximum force applied was limited to 18 N. Samples were then cooled to −5 °C and kept isothermally for 2 min to ensure shape fixing. Samples were unloaded at 0.03 N/min and heated back to 60 °C at 3 °C/min to measure shape recovery. This cycle was repeated thrice, and recovery ratio (*R*_r_) and fixity ratio (*R*_f_) were measured at each cycle using eqs 3 and 4, respectively, where ϵ_u_ is the strain after unloading (the fixed shape), ϵ_m_ is the maximum strain at loading, and ϵ_p_ is the remaining strain after recovery (permanent strain).
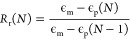
3
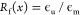
4

To test the shape memory properties of foams, samples (*n* = 3) were cut into cylinders (diameter = 6 mm, length = 1 cm), heated above their transition temperature, and crimped radially using a radial compression crimper (Blockwise Engineering, Tempe, AZ, USA) into a temporary low-profile geometry, A Nitinol wire (diameter = 3 mm) was passed through the foam samples to hold them in place, and foams were allowed to expand in a water bath at 37 °C. Images were captured using a camera at 5 s intervals over 10 min. Volume was measured at each interval using the diameter and the length of the cylinder in the images. Images were analyzed using ImageJ to quantify foam dimension over time, and % volume recovery was measured at each time point (*t*) according to eq 5. Volume recovery was plotted over the expansion time frame.
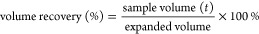
5

### 2.8 Mechanical Properties

To test compressive mechanical properties, 8 mm punches (*n* = 3) were cut from the foams and incubated with phenolic acid solutions overnight. Samples were then dried under a −1015 mbar vacuum at 40 °C for 2 days to ensure complete removal of DMSO. Samples were soaked in DI water at 50 °C for 3 h to allow them to swell. Control hydrogels without phenolic acids were simply swollen in DI water. Before testing, wet samples were removed from the water, lightly patted, and cut to ensure that the diameter to height ratio was maintained at 2:1. Samples were compressed using a 24 N load cell (Test Resources, Shakopee, MN, USA) until failure. Compressive modulus was measured from 0 to 2% strain.

### 2.9 Antibacterial Property Evaluation

*Escherichia coli* (*E. coli,* 397E strain, ATCC, Manassas, VA, USA) was used to test the antimicrobial efficacy of phenolic acid-containing hydrogels. Samples (n = 3) were punched from hydrogel films and sterilized via UV–C radiation for 3 h. Silver-based foam dressings (AREZA MEDICAL, Dallas, TX, USA) were cut to similar dimensions as the samples and served as positive (antimicrobial) controls. Hydrogels without phenolic acid incorporation served as negative controls. *E. coli* was cultured as previously described.^25^ Briefly, bacteria were incubated in 5 mL sterile lysogeny broth (LB, prepared at 25 g/L of deionized water and autoclaved) at 37 °C. After 16 h, 1 mL of the bacteria solution was transferred to 10 mL of fresh LB and incubated at 37 °C until the bacteria reached the logarithmic growth period, at which an optical density of 0.6 at an absorbance of 600 nm was achieved. The optical density was measured using a plate reader (FLx800, Bio-Tek Instruments, Inc.). Then, 100 μL of this bacteria solution was added to each well-containing sample and incubated for 1 h. The bacteria were diluted by a factor of 10^6^ using LB, and three 10 μL drops were pipetted onto an LB-agar plate from each well. Plates were incubated at 37 °C overnight. Images were captured at each drop location after 18 h, and colony forming unit (CFU) density was qualitatively assessed as a measure of sample antimicrobial properties as previously described.^26^

To characterize antimicrobial property retention, samples were incubated in PBS (2 mL per sample) at 37 °C for up to 30 days. PBS was replaced and stored for characterization of surrounding media every 10 days, and a set of samples were removed from PBS at 0, 10, 20, and 30 days for characterization of scaffold properties. The antimicrobial properties of all samples and surrounding media were measured together at the end of the 30-day study.

### 2.10 Phenolic Acid Release

**Figure 2:**
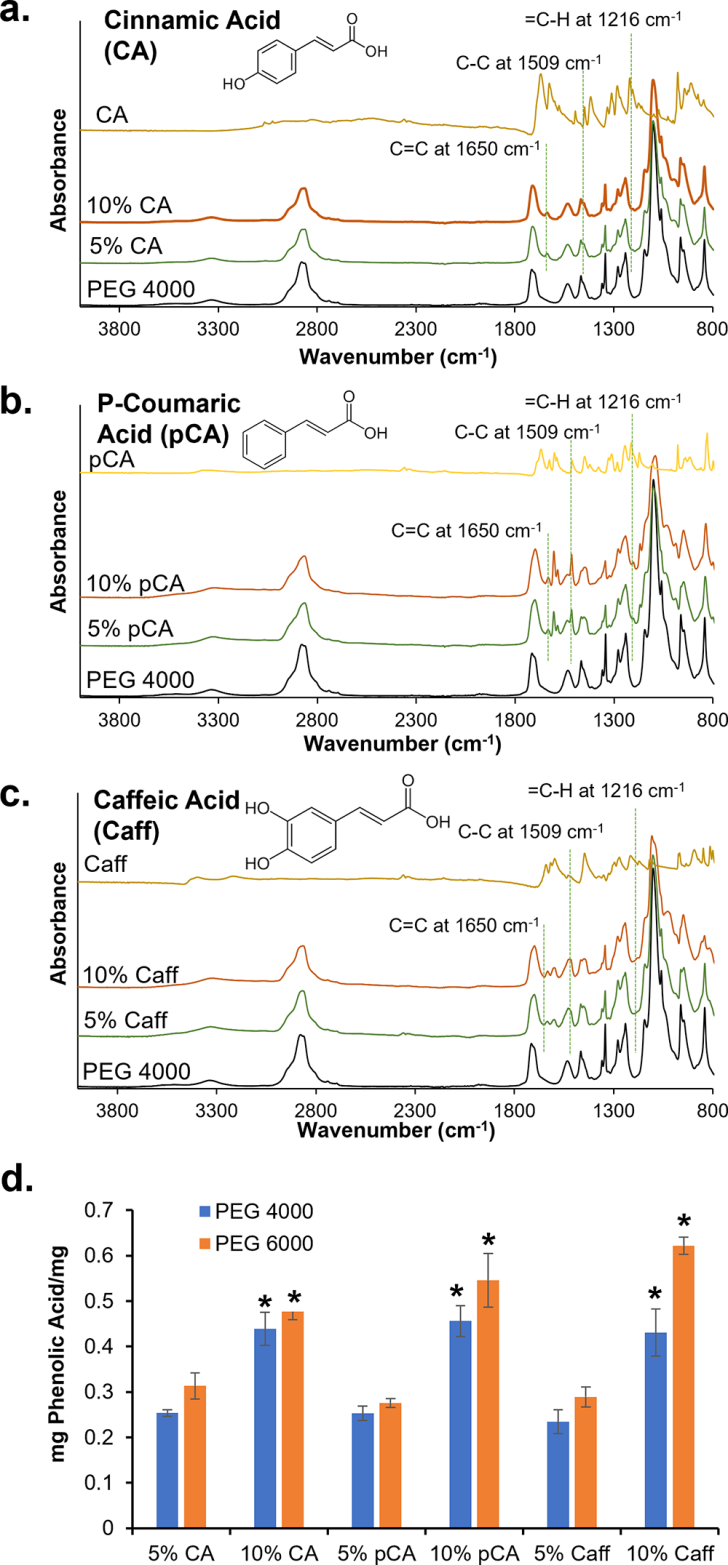
Fourier transform infrared spectroscopy of PEG 4000 hydrogel samples before and after physical incorporation of (a) cinnamic acid, (b) *p*-coumaric acid, and (c) caffeic acid. (d) Phenolic acid loading in PEG 4000 and PEG 6000 hydrogels. Mean ± standard deviation displayed. *n* = 3. *p < 0.05 between formulations under brackets.

Phenolic acid release from hydrogels was measured using a UV–vis spectrophotometer (Evolution 60, Fisher Scientific, Waltham, MA, USA). Phenolic acid concentrations were quantified using a reference peak (CA: 270 nm; Caff: 254, 274, and 384 nm; and pCA: 345 nm) to assess release rates over time. To measure release rates, samples with PAs were placed in a microcentrifuge tube containing 2 mL of 1X PBS and incubated at 37 °C. Triplicates were prepared for each sample. Separate samples were prepared for each time point −10, 20, and 30 days. At each time point, the sample was removed from the surrounding media to analyze the bacterial interaction mentioned in the earlier section. The tubes were then agitated via a vortex and 600 μL of PBS was removed to analyze bacterial interactions of the surrounding media. The remaining 1400 μL of PBS from each sample was diluted with 1400 μL DMSO to ensure the complete solubility of phenolic acids in the solution before measuring the PA content using a UV–vis spectrophotometer. The dilution level was taken into consideration while measuring PA release rates. The structure of each PA is shown in Figure 2a–c. Control hydrogels were stored in PBS for 1 week, and UV–vis was employed on the surrounding media to ensure that no hydrogel leachables could contribute to the PA release measurement. No measurable absorbance values were obtained with the controls at the wavelength of interest.

### 2.11 Cytocompatibility

Cytocompatibility of samples was measured using 3T3 Swiss mouse fibroblasts (ATCC-CL92; ATCC, Manassas, VA, USA). Cells were cultured in Dulbecco’s modified Eagle’s medium supplemented with 10% heat-inactivated fetal bovine serum (FBS) and 1% penicillin-streptomycin (P/S, Gibco) at 37 °C/5% CO_2_ for 24 h). Cells were used at passage 13 after 3 days of culture and seeded onto a 24-well tissue culture polystyrene plate at 10,000 cells/well for 24 h at 37 °C, 5% CO_2_. To test the effects of phenolic acids, hydrogel samples were soaked in phenolic acid solutions for 24 h at 50 °C, cut using a 6 mm biopsy punch while swollen, vacuum-dried at 40 °C and −1015 mbar for 72 h, and sterilized via UV–C radiation sterilizer (UV sterilizer and sanitizer cabinet, Skin Act, Pacoima, CA, USA) for 3 h. Sterilized samples were placed in 0.4 μm Transwell inserts above cells seeded in 24 well plates to measure indirect cytocompatibility of samples. Cells were incubated with samples (n = 3) at 37 °C, 5% CO_2_ for 24 h. Cells incubated without samples were used as positive (cytocompatible) controls, and cells exposed to 200 μL of 70% methanol for 24 h were used as negative (cytotoxic) controls. Cytocompatibility was assessed via Live/Dead assay (ThermoFisher Scientific, Waltham, MA, USA). Cells were stained with green-fluorescent calcein-AM (live cells) and red-fluorescent ethidium homodimer-1 (dead cells) for 15 min at 37 °C while covered with aluminum foil to protect cells from direct light exposure. Cells were imaged via an inverted microscope (Leica, DMI6000) at 10× magnification to determine the number of live (green) and dead (red) cells. Three images were captured per sample well. Cytocompatibility was measured according to eq 6. Additionally, resazurin assay was used to assess cytocompatibility of PEG 4000 and PEG 6000 hydrogels with and without phenolic acids incorporation over a period of 1 week. Cytocompatibility was measured based on the fluorescence emission 570 nm using a plate reader (FLx800, BioTek Instruments, Inc.) as per eq 7.
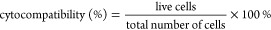
6
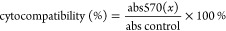
7

### 2.12 Statistical Analysis

Measurements are presented as mean ± standard deviation. The number of measurements was maintained at three for all analysis. Student’s *t* tests (2-sample, assuming unequal variance) were performed between controls and phenolic acid hydrogels as mentioned in each figure legend. For comparisons between multiple groups, ANOVA with Tukey’s post hoc was performed. Statistical significance was accepted as *p* < 0.05.

## 3 Results

### 3.1 Gel Fraction and Swelling Ratio

Both polymer compositions had gel fractions above 96% indicating a complete reaction of a highly cross-linked polymer network, Table 1. In general, swelling ratios of PEG 4000 hydrogels were approximately double the initial dry weight, with swollen PEG 6000 hydrogels swelling more than 2.5 times their original dry weight.

### 3.2 Phenolic Acid Incorporation

Successful phenolic acid absorption was confirmed via the presence of characteristic peaks corresponding to phenolic rings of CA, pCA, and Caff in the FTIR spectra (C=C at ∼1650 cm^–1^, C–C at ∼1509 cm^–1^, and =C–H at ∼1216 cm^–1^), which are not visible in the control PEG hydrogels. Representative spectra of PEG 4000 hydrogels are shown in Figure 2a–c; similar observations were made in the PEG 6000 spectra before and after phenolic acid incorporation. Physical absorption of phenolic acids from the 10% solutions was statistically higher compared to the respective 5% solutions, Figure 2d. A general increase in phenolic acid absorption was also observed with increased PEG molecular weight in PEG 6000 hydrogels.

### 3.3 Mechanical Properties

**Figure 3:**
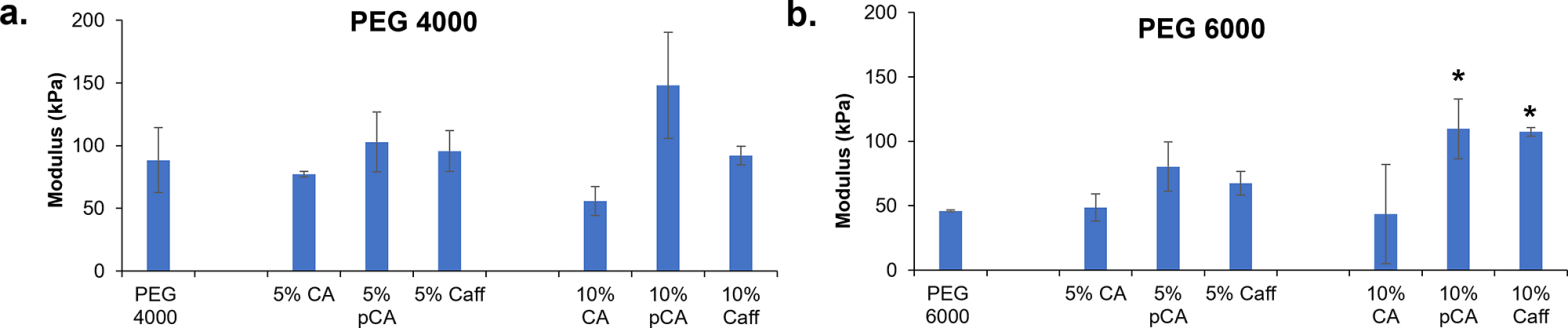
Compressive modulus of (a) PEG 4000 and (b) PEG 6000 hydrogels in the wet state before and after physical incorporation of phenolic acids. Mean ± standard deviation displayed. *n* = 3. *p < 0.05 relative to PEG control without phenolic acid incorporation.

Compressive modulus was measured on foams with 70% porosity (described in section 2.2) before and after phenolic acid incorporation, Figure 3. Overall, compressive modulus values range from 88 to 148 kPa for PEG 4000-based hydrogels and from 46 to 107 kPa for PEG 6000-based hydrogels. A general decrease in compressive modulus with increased CA content was observed, whereas pCA incorporation increased compressive modulus. Caff incorporation did not significantly alter the compressive modulus of PEG 4000 hydrogels, but an increase in compressive modulus is seen with increased Caff content in PEG 6000 hydrogels.

### 3.4 Thermal Properties

**Figure 4:**
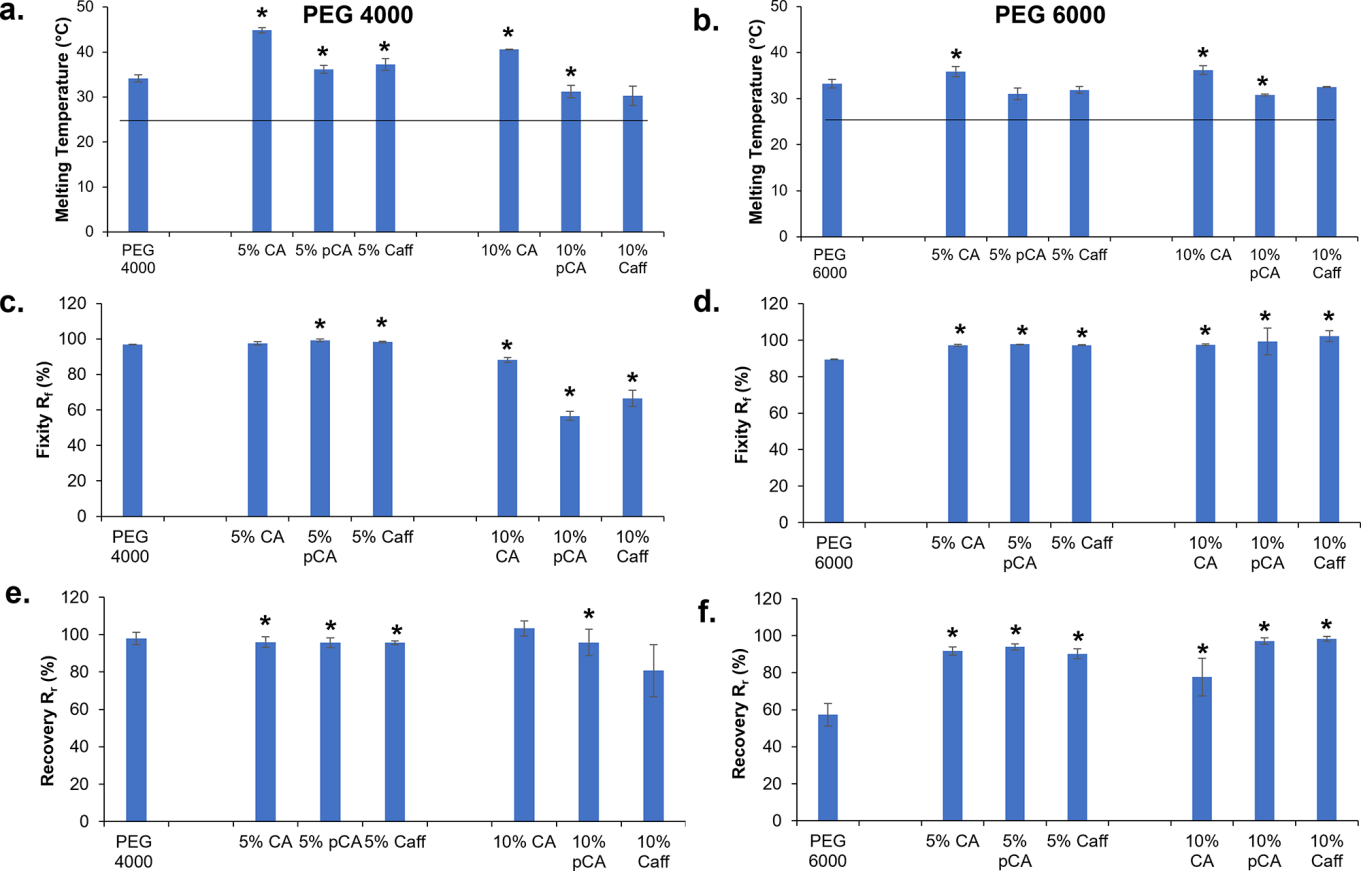
Melting temperatures of (a) PEG 4000 and (b) PEG 6000 hydrogels before and after phenolic acid incorporation were measured using differential scanning calorimetry. The horizontal lines denote room temperature to indicate potential for shape fixity during storage. Shape memory properties (fixity: (c,d) and recovery: (e,f)) of (c) and (e) PEG 4000 and (d) and (f) PEG 6000 hydrogels before and after phenolic acid incorporation measured using dynamic mechanical analysis. *p < 0.05 relative to PEG control without phenolic acid incorporation.

All hydrogels had *T*_m_’s between 30 and 40 °C, Figure 4a,b. Slight increases in *T*_m_ were observed after cinnamic acid incorporation in both formulations. An overall reduction in *T*_m_ occurs as the phenolic acid content increases from 5% to 10%, with larger variations in *T*_m_ observed in PEG 4000 hydrogels compared to PEG 6000 hydrogels.

### 3.5 Shape Memory Properties

Dynamic mechanical analysis was used to measure the shape memory properties of hydrogel networks, Figure 4c–f. Shape fixity and recovery of PEG 4000 controls were high (>92%) and were slightly increased after 5% phenolic acid incorporation. Higher phenolic acid content PEG 4000 hydrogels displayed a general reduction in shape fixity and shape recovery, with larger differences observed in 10% pCA and 10% Caff hydrogels. Shape fixity was high (∼90%) for all PEG 6000 hydrogels, and phenolic acid incorporation increased fixity, with minimal variations based on phenolic acid content or type. PEG 6000 control hydrogels had relatively low shape recovery (57%), which was increased to >80% after phenolic acid incorporation at low and high concentrations.

### 3.6 Antimicrobial Properties

**Figure 5:**
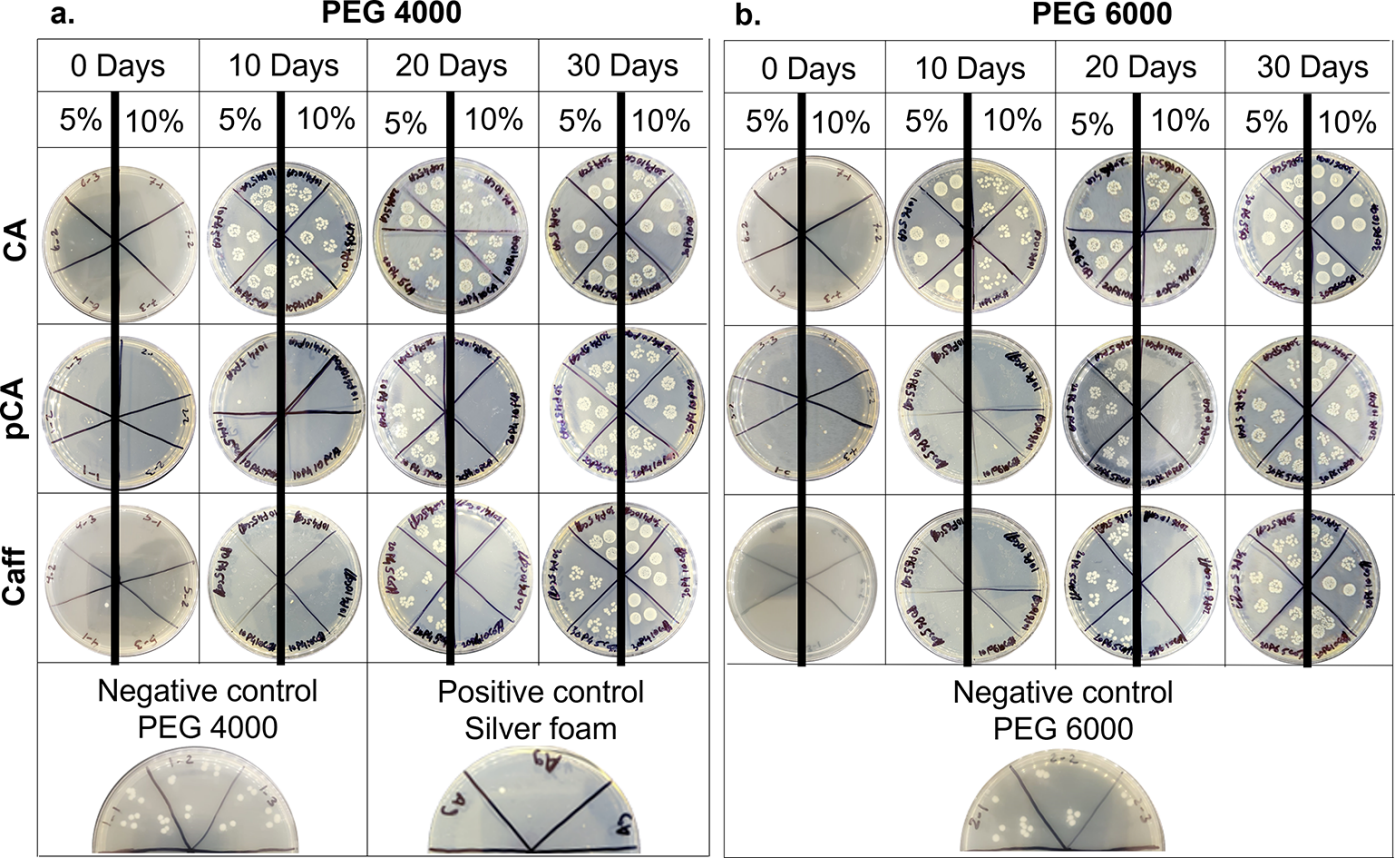
Antimicrobial properties of (a) PEG 4000 and (b) PEG 6000 hydrogels before and after physical incorporation of phenolic acids over 30 days of storage in phosphate-buffered saline. The left side denotes 5% and the right side denotes 10% phenolic acid incorporated into the respective hydrogel controls, shown in the lowest row. Images of colony forming units are displayed.

**Figure 6:**
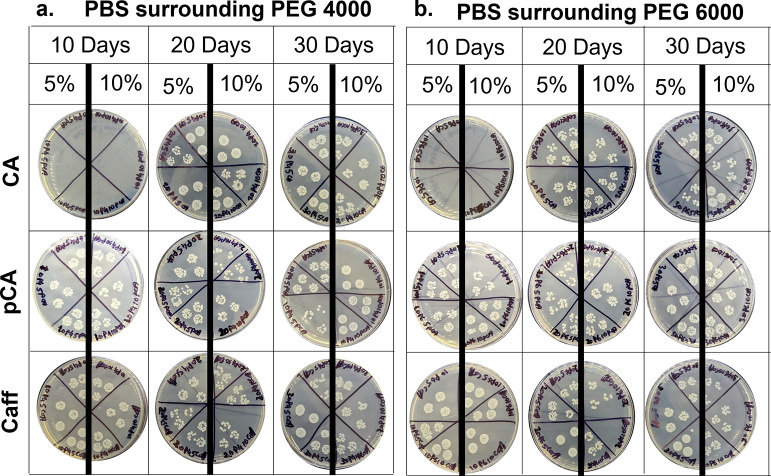
Antimicrobial properties of surrounding PBS solutions in which phenolic acid-containing (a) PEG 4000 and (b) PEG 6000 hydrogels were incubated. Solutions were tested at 10, 20, and 30 days. Images of colony forming units are displayed.

CA hydrogels had initially high antimicrobial properties, as evidenced by low CFU counts at 0 days of incubation in PBS, Figure 5. Antimicrobial properties were quickly diminished by 10 days, when high numbers of CFUs were present. CFU density generally increased over 20–30 days of CA hydrogel incubation in PBS. The pCA and Caff hydrogels had excellent antimicrobial properties on days 0 and 10, with minimal CFUs present, Figure 6. The 10% pCA and Caff hydrogels retained antimicrobial properties at 20 days, whereas an increase in CFUs was observed in the 5% pCA and Caff hydrogels. By 30 days, all hydrogels had negligible antimicrobial properties.

Upon analyzing the antimicrobial properties of the surrounding PBS solutions, it was observed that CA hydrogel solutions had negligible CFUs at 10 days. CFU densities were generally high in PBS surrounding CA hydrogels after 20 and 30 days, indicating that the majority of CA was released within 10 days, Figure 6. PBS from the other hydrogels at 10 days did not inhibit CFU formation. There was a slight reduction in CFUs observed in PBS surrounding pCA and Caff hydrogels at 20 days. An increase in CFUs was observed at 30 days in all hydrogel solutions. No clear trends were observed between the antimicrobial properties of corollary PEG 4000 and PEG 6000 formulations.

### 3.7 Phenolic Acid Release

**Figure 7:**
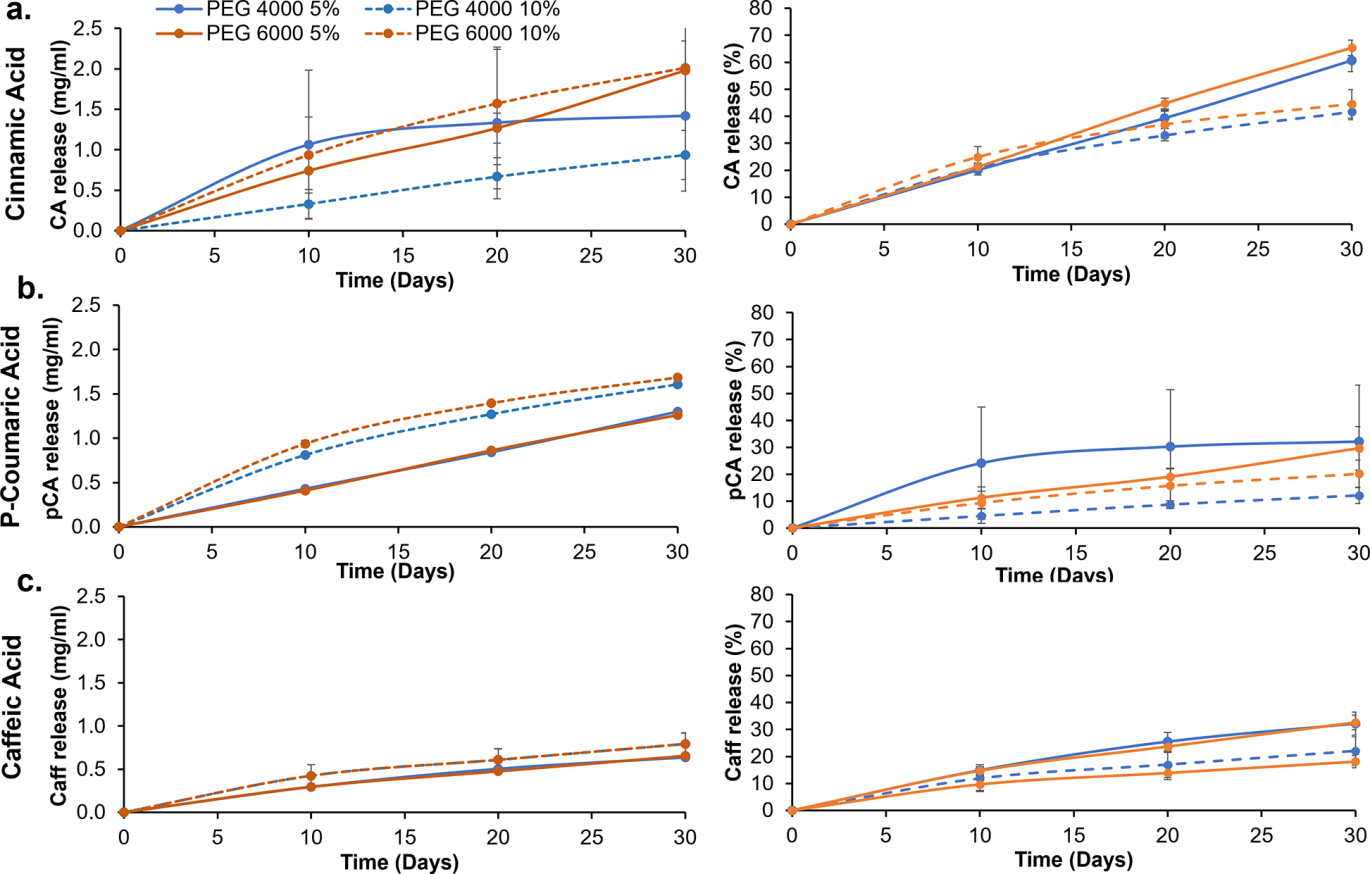
Quantification of (a) cinnamic acid, (b) *p*-coumaric acid, and (c) caffeic acid released from the hydrogel networks over 30 days measured using UV–vis spectroscopy. Quantifications are provided in mg phenolic acid/ml of PBS (left column) and percent release relative to initial incorporation (right column). Mean ± standard deviation displayed. *n* = 3. Legend in top left applies to all charts.

As seen in Figure 7, an initial release was observed during the first 10 days across all hydrogel formulations. Between 10 and 20 days, the phenolic acid release was slower compared to the first 10 days, and minimal additional release was observed between 20 and 30 days. Overall, increased phenolic acid release was observed among hydrogels with higher phenolic acid content (10% vs 5%) and higher PEG molecular weight (PEG 6000 vs PEG 4000). Additionally, as the number of ring hydroxyls was increased on the phenolic acids (CA < pCA < Caff), lower release rates were observed.

### 3.8 Cytocompatibility

**Figure 8:**
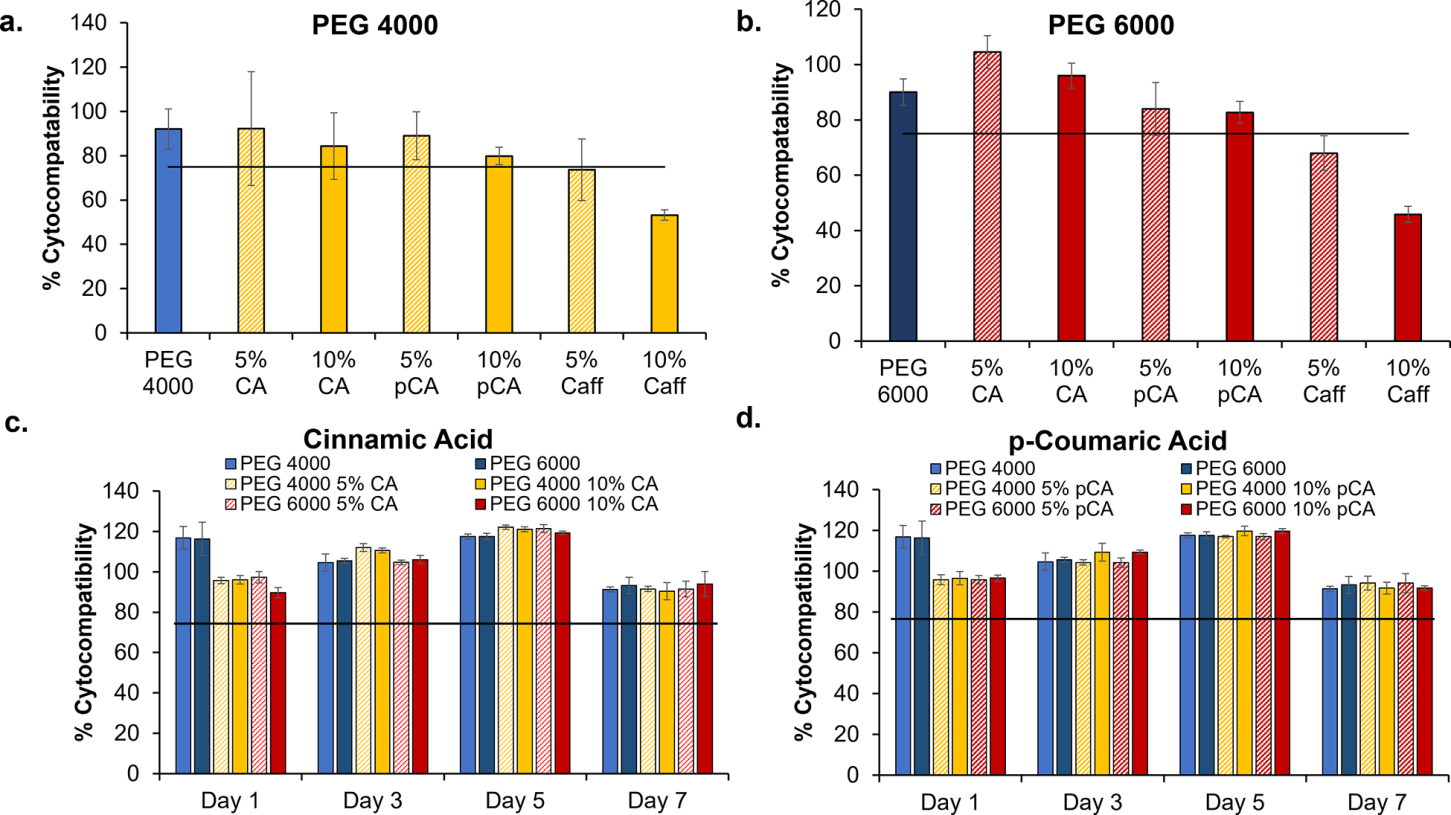
Cytocompatibility of 3T3 mouse fibroblast cells over 24 h in the presence of (a) PEG 4000 and (b) PEG 6000 hydrogels before and after the physical incorporation of phenolic acids. Cytocompatibility of 3T3 mouse fibroblasts over 1 week in the presence of (c) cinnamic acid and (d) *p*-coumaric acid containing hydrogels. Mean ± standard deviation displayed. *n* = 3. The horizontal line denotes the ISO standard (75% cytocompatibility). No statistical significance observed among samples over 1 week within the same group.

Control hydrogels had high cytocompatibility (>90%) over 24 h, Figure 8. In general, cytocompatibility was reduced with an increase in phenolic acid content from 5% to 10%. The overall cytocompatibility of CA and pCA hydrogels was maintained above the ISO – 10993 standards (>75% cytocompatibility).^27^ However, Caff hydrogels had lower cytocompatibility below 75%, with larger reductions in cytocompatibility (<50%) observed in hydrogels with 10% Caff content. When tested over 1 week period, both CA (Figure 8c) and pCA (Figure 8d) containing hydrogels exhibited satisfactory cytocompatibility.

### 3.9 Volume Recovery of Foams

**Figure 9:**
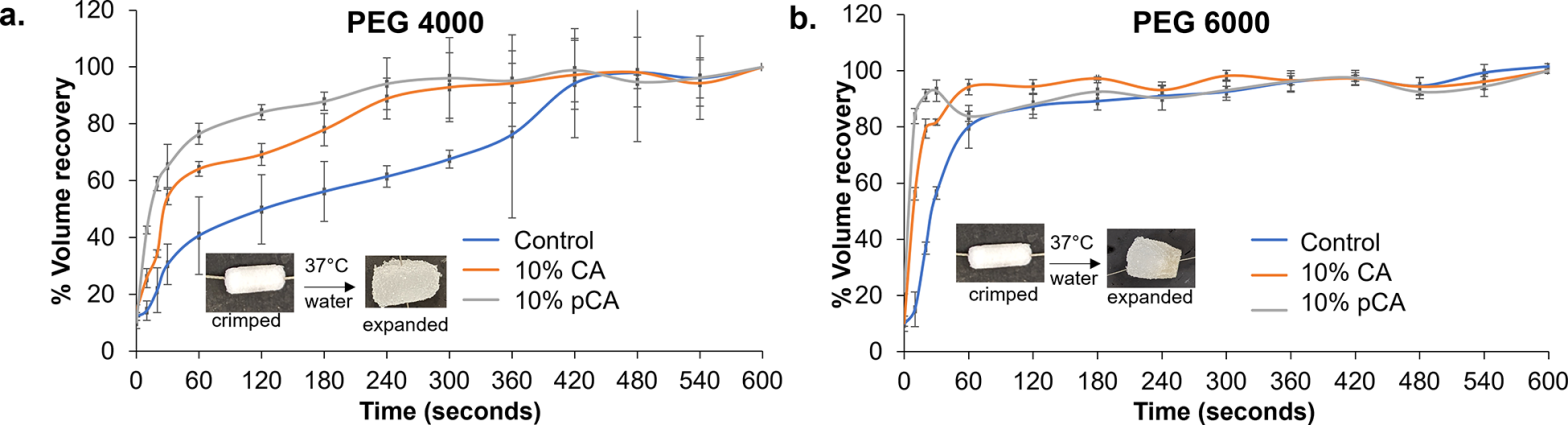
Volume recovery of (a) PEG 4000 and (b) PEG 6000 control foams compared with foams with cinnamic acid and p-coumaric acid. Mean ± standard deviation displayed. *n* = 3.

Upon identification of hydrogels that had antimicrobial properties and cytocompatibility, volume recovery of radially crimped foams was characterized in 37 °C water as an initial indication of foam dressing expansion in the body, Figure 9. Incorporating phenolic acids resulted in a faster volume recovery in both PEG 4000 and PEG 6000 foams. PEG 4000 foams generally had slower expansion, with controls reaching maximum volume recovery at ∼6 min, Figure 9a. Inclusion of 10% CA and 10% pCA increased PEG 4000 volume expansion rates to reach a maximum volume within ∼4 min. PEG 6000 control foams recovered very rapidly and took ∼30 s to recover 100% of their original volume. PEG 6000 foams with 10% CA and 10% pCA recovered 100% of their original volume within 5 to 15 s, as seen in Figure 9b.

## 4 Discussion

This work describes a new shape memory polymer hydrogel system. We demonstrate the capability to simultaneously improve shape memory properties and impart antimicrobial capabilities to a polymer network postfabrication. This modification approach can be applied to any polymer system that has the potential to form hydrogen bonds with phenolic acids to enable physical incorporation into the network. Furthermore, the system could be employed with other small molecule drugs of interest that contain hydrogen bonding sites.

The increased swelling ratio among the PEG 6000 hydrogels can be attributed to a longer polymer chain length, which results in relatively lower cross-link density and increased water absorption.^20^ The increased swelling ratio in PEG 6000 hydrogels correlated with increased phenolic acid absorption. This result was expected, as the phenolic acids get absorbed onto the hydrogels via diffusion during swelling. It is hypothesized that phenolic acids are stabilized by hydrogen bonding between the urethane groups within the polymer network and the hydroxyl groups on the phenolic acids (i.e., carboxylic acid end groups of all three phenolic acids and hydroxyls on phenolic rings of pCA and Caff).

The reported elastic modulus of skin is between 420 and 850 kPa;^28,29^ an overall compressive modulus of the SMP hydrogels below 150 kPa ensures that hydrogel wound dressings would not impart excessive stress to the surrounding wound walls postswelling. The lower compressive modulus of PEG 6000 hydrogels can be attributed to a longer monomer length that results in a lower cross-link density.^30^ These mechanical property measurements were taken on water-swollen hydrogels, in which the hydrogen bonds between urethane groups in the polymer network are plasticized.^31^ A general increase in compressive modulus after the physical incorporation of phenolic acids, particularly in the PEG 6000 hydrogels, is attributed to hydrogen bonds between phenolic acids and the polymer network to increase physical cross-linking. Therefore, phenolic acid incorporation provides a simple tool for tuning hydrogel modulus independently of network chemistry. While the focus of this work was not on finely tuning scaffold stiffness, this concept could be applied to any biomaterial system with hydrogen bonding sites as a new method for altering modulus.

The new intermolecular hydrogen bonds formed between the hydroxyl and urethane groups disrupt the regular hydrogen bonds of the polyurethane network, which can alter the melting temperatures. The changes in melting temperature were larger in the PEG 4000 hydrogels, which we hypothesize is due to the shorter PEG chains that cannot form crystals as readily.^20^ In these gels, small amounts of phenolic acids acted as cross-linkers between chains, increasing crystal stability and corollary *T*_m_’s. Higher concentrations of phenolic acids with multiple hydrogen bonding sites (pCA and Caff) had the opposite effect and reduced *T*_m_; in these hydrogels, the phenolic acids act more like plasticizers, separating polymer chains and reducing crystal stability. Similar trends can be seen in the PEG 6000 hydrogels, but the overall effects of phenolic acids on *T*_m_ were reduced in the PEG 6000 formulations.

The synthesized polyurethane hydrogels demonstrate shape memory properties around their *T*_m_. All measured *T*_m_’s were above room temperature, and shape fixity was high across all formulations. In general, trends in shape fixity and recovery of PEG 4000 hydrogels after phenolic acid incorporation matched trends in *T*_m_, with increased shape memory properties after incorporation of low amounts of phenolic acids and reductions in shape memory properties in hydrogels with higher concentrations of pCA and Caff. PEG 6000 hydrogels all exhibited increases in shape fixity and recovery after phenolic acid incorporation, with larger increases observed with higher numbers of hydrogen bonding sites on the phenolic acids. This result indicates that phenolic acids act more like cross-linkers in the PEG 6000 hydrogels, stabilizing the temporary shape and enabling faster and more complete recovery to the primary shape.

This system provides a novel mechanism for tuning thermal and shape memory properties of polymer networks that could be applied to a range of shape memory polymers. Higher thermal transitions and shape fixity enables fixation and storage in the temporary shape at room temperature without premature recovery before implantation. Improvements in shape recovery would enable expansion to the permanent shape after implantation to fill wounds with dressing materials. When these materials are exposed to water, hydrogen bonds that stabilize the secondary shapes are plasticized to soften the hydrogel and enable shape recovery at lower temperatures. This effect was seen in Figure 9, where hydrogel foams expanded to their primary shape in 37 °C water. Furthermore, 10% CA and 10% pCA foams had a faster volume recovery due to the plasticization of urethane linkages by the phenolic acids. These shape recovery properties could be harnessed for wound filling in future work. Dry, compressed hydrogels could be applied to wound beds, where they would expand after heating to body temperature to fill irregular wound shapes. Then, the hydrogels would swell to maintain a moist wound environment.

All phenolic acid hydrogels had initially high antimicrobial properties with minimal CFUs compared with negative controls. Retention of antimicrobial properties over time of incubation in PBS was dependent on the number of hydrogen bonding between phenolic acids and the polyurethane hydrogel network. Namely, more rapid loss of antimicrobial properties in CA hydrogels can be attributed to the absence of free hydroxyls on the phenolic ring of CA, which limits strong intermolecular bonding with the polymer network and allows CA to be more easily released. These release results were corroborated by the high antimicrobial properties of surrounding PBS at 10 days and the relatively large amount of CA released in this time frame.

Hydrogels with pCA and Caff better retained antimicrobial properties, particularly in higher phenolic acid content samples; negligible CFUs were observed in 10% pCA and Caff hydrogels at 20 days, while 5% pCA and Caff hydrogels had significantly more CFUs at 20 days in comparison with corollary 10% samples. By 30 days, CFU counts were high after incubation with all hydrogels, indicating that the concentration of phenolic acids remaining in the hydrogels at this time point was not sufficient for imparting antimicrobial efficacy to the hydrogels.

At 10 days, the pCA and Caff solutions had high CFU densities. It is hypothesized that the amount of pCA and Caff released in the first 10 days was too low to affect the antimicrobial properties of the surrounding solutions. On day 20, there are slightly lower densities of CFUs among the pCA and Caff solutions compared to the day 10 results, indicating that the amount of pCa and Caff released between 10 and 20 days imparted surrounding media with antimicrobial properties. The phenolic acid release slowed after 20 days, and thus minimal release occurred between days 20 and 30. A high density of CFUs was observed after incubation of bacteria with surrounding PBS on day 30. The IC50 values for CA, pCA, and Caff against *E. coli* at 24 h were previously measured to be 2 to 3 mg/mL.^32^ The net amount of phenolic acid release between 20 and 30 days was below this IC50 value, substantiating the observed increases in CFU formation at 30 days. It is also possible that the phenolic acids were not stable within this time frame and that they experienced a loss in antimicrobial activity over time to reduce effects on surrounding media. This point warrants further investigation in future work.

In general, pCA and Caff had slower release profiles, which is attributed to their higher number of hydrogen bonding sites. These release results provide a rational framework for the selection of phenolic acids based on the number of hydrogen bonding sites. If an earlier release is desired, phenolic acids with fewer intermolecular interaction sites (e.g., CA) should be selected, while phenolic acids with more hydroxyl groups (e.g., pCA and Caff) would be preferred for long-term antimicrobial properties.

The high cytocompatibility of CA and pCA hydrogels provides a preliminary indication that these materials could serve as wound dressing materials. The low cytocompatibility of Caff hydrogels echoes previously obtained results wherein the cytocompatibility of Caff solutions dropped significantly over 24 h.^25^ Thus, Caff may not be an ideal candidate for use in wound dressings. In our previous work, the silver dressing that was used as a positive control in the antimicrobial testing had very low cytocompatibility (30%).^33^ The results in this study indicate that CA and pCA provide options for the addition of antimicrobial properties into hydrogels to reduce infection risks without affecting surrounding mammalian cells.

Apart from the observed improvements in shape memory and antimicrobial properties, phenolic acids also have several other functionalities that may be beneficial in wound healing. For example, phenolic acids are antioxidants that can scavenge reactive oxygen species (ROS) by inhibiting ROS-generating enzymes and chelation with ROS-forming ferrous (Fe^2+^) ions.^34^ The reduction of ROS could aid the chronic wound healing process and reduce chronic inflammation that is caused by excessive ROS.^35−37^ In general, these hydrogels provide a promising platform for future development as chronic wound dressing materials. Additionally, this work provides novel methods to tune hydrogel properties without changing the overall network chemistry.

## 5 Conclusions

This study presents a simple technique to tune polymer structures independently of overall network chemistry, impart easy-to-control antimicrobial properties, and improve shape memory properties postfabrication. The number of hydroxyl groups present in the incorporated phenolic acids influences the extent to which the material properties can be tuned (i.e., whether incorporated phenolic acids act more as cross-linkers or plasticizers). These considerations could be applied to the incorporation of other drugs/bioactive agents into biomaterials via hydrogen bonding to control release while tuning material properties. The polymer molecular weight also affected the extent to which the properties could be altered, providing an additional level of control. These hydrogel materials have appropriate thermal properties to enable stable storage in the low-profile shape and fast actuation after implantation to rapidly fill wounds. Improved shape recovery after phenolic acid incorporation could ensure that wounds are completely sealed. Increased phenolic acid content and intermolecular interaction sites allowed for longer antimicrobial property retention, providing a framework for the selection of phenolic acids based on desired antimicrobial time frames. Finally, appropriate cytocompatibility indicates that these hydrogels may be suitable for future development as chronic wound dressings that reduce infection risks. Based on the data collected here, PEG 6000 hydrogels with 10% pCA has the best potential for use for chronic wound treatment due to their favorable shape memory properties, cytocompatibility, and sustained antimicrobial efficacy.

## Supporting Information Available

Thermogravimetric analysis curves of synthesized hydrogels (PDF)
