# Supplementary material for: Antimicrobial Shape Memory Polymer Hydrogels for Chronic Wound Dressings
Source: ACS Appl Bio Mater. 2022 Oct 18;5(11):5199–209. doi: 10.1021/acsabm.2c00617 (PMC9682482; doi:10.1021/acsabm.2c00617)
Supplement: Supplementary file 1 — mt2c00617_si_001.pdf [file mt2c00617_si_001.pdf]

Supporting Information

Antimicrobial shape memory polymer hydrogels for chronic wound dressings

Anand Utpal Vakil[1], Maryam Ramezani[1], and Mary Beth B. Monroe[1].*

[1]Department of Biomedical and Chemical Engineering, Syracuse Biomaterials Institute, and BioInspired Syracuse: Institute for Material and Living Systems, Syracuse University, Syracuse, NY 13244.

*Corresponding author: Dr. Mary Beth Browning Monroe

Department of Biomedical and Chemical Engineering, BioInspired Syracuse: Institute for Materials and Living Systems

Syracuse University

318 Bowne Hall

Syracuse, NY 13244

Tel: (315) 443-3323

E-mail: mbmonroe@syr.edu

Supplementary Data

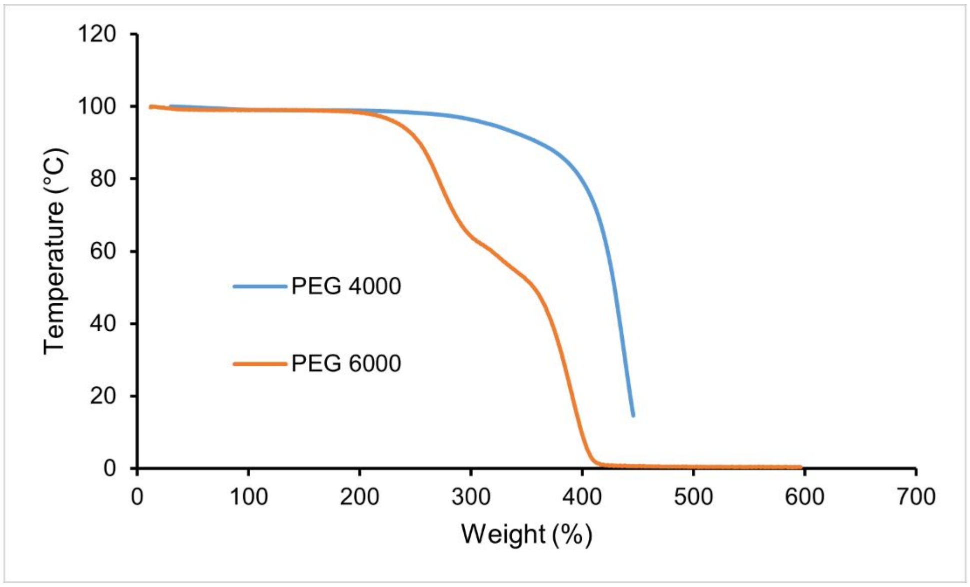

Figure S1. TGA curves of PEG 4000 and PEG 6000 hydrogels that depict 0.5% mass loss at temperatures greater than 200 °C.
